# Transducin Partners Outside the Phototransduction Pathway

**DOI:** 10.3389/fncel.2020.589494

**Published:** 2020-10-14

**Authors:** Dhiraj Srivastava, Ravi P. Yadav, Shivangi M. Inamdar, Zhen Huang, Maxim Sokolov, Kimberly Boyd, Nikolai O. Artemyev

**Affiliations:** ^1^Department of Molecular Physiology and Biophysics, The University of Iowa Carver College of Medicine, Iowa City, IA, United States; ^2^Department of Neurology and Neuroscience, University of Wisconsin-Madison, Madison, WI, United States; ^3^Department of Ophthalmology, Biochemistry and Neuroscience, West Virginia University, Morgantown, WV, United States; ^4^Department of Ophthalmology and Visual Sciences, Ophthalmology and Visual Sciences, The University of Iowa Carver College of Medicine, Iowa City, IA, United States

**Keywords:** retina, G protein, GPCR, transducin, Ric-8A, GEF, chaperone

## Abstract

Transducin mediates signal transduction in a classical G protein-coupled receptor (GPCR) phototransduction cascade. Interactions of transducin with the receptor and the effector molecules had been extensively investigated and are currently defined at the atomic level. However, partners and functions of rod transducin α (Gα_t__1_) and βγ (Gβ_1_γ_1_) outside the visual pathway are not well-understood. In particular, light-induced redistribution of rod transducin from the outer segment to the inner segment and synaptic terminal (IS/ST) allows Gα_t1_ and/or Gβ_1_γ_1_ to modulate synaptic transmission from rods to rod bipolar cells (RBCs). Protein-protein interactions underlying this modulation are largely unknown. We discuss known interactors of transducin in the rod IS/ST compartment and potential pathways leading to the synaptic effects of light-dispersed Gα_t1_ and Gβ_1_γ_1_. Furthermore, we show that a prominent non-GPCR guanine nucleotide exchange factor (GEF) and a chaperone of Gα subunits, resistance to inhibitors of cholinesterase 8A (Ric-8A) protein, is expressed throughout the retina including photoreceptor cells. Recent structures of Ric-8A alone and in complexes with Gα subunits have illuminated the structural underpinnings of the Ric-8A activities. We generated a mouse model with conditional knockout of Ric-8A in rods in order to begin defining the functional roles of the protein in rod photoreceptors and the retina. Our analysis suggests that Ric-8A is not an obligate chaperone of Gα_t1_. Further research is needed to investigate probable roles of Ric-8A as a GEF, trafficking chaperone, or a mediator of the synaptic effects of Gα_t1_.

## Introduction

A prototypical heterotrimeric G protein, transducin (Gα_t_βγ), is a key signaling mediator in the visual transduction cascade in vertebrate rod and cone photoreceptors. Traditionally, studies of transducin focused on its structure and mechanisms underlying this signaling cascade. Phototransduction takes place in a specialized ciliary compartment of photoreceptor cells called the outer segment (OS). Absorption of light by rhodopsin allows the photoexcited receptor (R^∗^) to stimulate GDP/GTP exchange on Gα_t_βγ, thus releasing Gα_t_GTP from Gβγ and R^∗^. Subsequent activation of cGMP phosphodiesterase 6 (PDE6) by Gα_t_GTP causes concentration of cytoplasmic cGMP to drop, halting conductance of cGMP-gated channels in the plasma membrane (Arshavsky et al., 2002; Fu and Yau, 2007). Besides rhodopsin and PDE6, an essential partner of transducin in the visual cascade is a photoreceptor-specific member of the RGS (regulators of G protein signaling) family, RGS9-1. RGS9-1 in complex with Gβ_5L_ acts as a GTPase-activating protein for Gα_t_, and thus is a major regulator of the turn-off of the visual signal (He et al., 1998, 2000; Makino et al., 1999; Cowan et al., 2001; Martemyanov and Arshavsky, 2009; Arshavsky and Burns, 2012). Together, the interactions of transducin with R^∗^, PDE6, and RGS9-1/Gβ_5L_ control the signal amplification, sensitivity, and speed of photoresponses. The remarkable molecular level of insight into these interactions has been recently elevated with solutions of the cryo-EM structures of transducin complexed with rhodopsin and PDE6 (Gao et al., 2019, 2020). Nevertheless, several important aspects of transducin biology, including its folding, trafficking, and roles outside the phototransduction cascade remain largely obscure. Rod transducin subunits Gα_t1_ and Gβ_1_γ_1_ are known to undergo bi-directional translocation between the OS and inner compartments of rod photoreceptors in a light-dependent manner, but its partners in the inner photoreceptor compartments, the inner segment and synaptic terminal (IS/ST), and the significance of these interactions are not well understood (Sokolov et al., 2002; Calvert et al., 2006; Artemyev, 2008; Slepak and Hurley, 2008). Light-induced translocation of Gα_t1_ and Gβ_1_γ_1_ was shown to reduce phototransduction gain, and thus it contributes to light adaptation of photoreceptor cells (Sokolov et al., 2002). Analyses of mouse models with impaired transducin translocation also support an important role of the phenomenon in neuroprotection of rods, presumably by reducing the metabolic stress associated with the constitutive phototransduction reactions under light conditions saturating responsiveness of rods (Fain, 2006; Peng et al., 2011; Majumder et al., 2013; Tian et al., 2014). Arguably, the most intriguing and poorly understood consequence of Gα_t1_ and Gβ_1_γ_1_ translocation is its modulation of the synaptic transmission from rods to rod bipolar cells (RBCs). Block of transducin translocation via additional lipid anchoring of the Gα_t1_ A3C mutant in a mouse model desensitized signal transmission to RBC, suggesting that transducin translocation enhances signaling to RBC in wild type mice (Majumder et al., 2013). Ultimately, the mechanism underlying the synaptic effect of transducin may involve its interactions with the synaptic machinery and/or modulation of the voltage-gated Ca^2+^ channels, Ca_v_1.4. Analysis of known and identification of novel partners of transducin in the IS/ST may provide clues to this mechanism.

## Transducin Partners in the Inner Photoreceptor Compartments: UNC119 and LGN/GPSM2

One well-known partner of Gα_t1_ outside the phototransduction cascade is Uncoordinated 119 (UNC119). UNC119 is a mammalian ortholog of *C. elegans unc-119* (Maduro and Pilgrim, 1995), also known as Retina Gene 4 protein (RG4; Higashide et al., 1996). UNC119 is uniquely abundant in photoreceptor cells, specifically the rod IS/ST, and its levels in other tissues are significantly lower (Higashide et al., 1998; Swanson et al., 1998; Karim et al., 2010). Truncation mutation in UNC119 is linked to cone-rod dystrophy in human patients (Kobayashi et al., 2000), whereas knockout of UNC119 in mice causes slow retinal degeneration (Ishiba et al., 2007). UNC119 shares sequence and structural homology with the prenyl-binding protein PDE6δ (or PDE6D), both featuring immunoglobulin-like β-sandwich fold serving as a lipid-binding module (Zhang et al., 2011). Unlike PDE6δ, which is a prenyl-binding protein, UNC119 selectively binds myristoyl moiety (Zhang et al., 2004, 2011; Gopalakrishna et al., 2011). Owing its lipid-binding specificity, UNC119 is generally viewed as a carrier protein for myristoylated cargo, with a preference for cargo proteins targeted to primary cilia via an ARL3-dependent mechanism (Ismail et al., 2012; Fansa and Wittinghofer, 2016; Frederick et al., 2020). Consistent with the role of UNC119 as a trafficking chaperone of transducin, the anterograde transport of translocated Gα_t1_ to the OS during dark adaptation is impaired in UNC119 knockout mice (Zhang et al., 2011). Quantification of UNC119 in rods suggests that the light-dispersed Gα_t__1_ is a major partner of UNC119 (Sinha et al., 2013).

However, the function of UNC119 in the retina appears to extend beyond that of a trafficking chaperone across the connecting cilium. Besides Gα_t1_, UNC119 interacts in a lipid modification-independent manner with CaBP4 (Haeseleer, 2008). Notably, the levels of UNC119 are reduced in mice lacking Gα_t1_ or CaBP4, suggesting that UNC119 functionally interacts with both of these proteins (Haeseleer, 2008; Sinha et al., 2013). CaBP4 is an EF-hand Ca^2+^ binding protein that binds to and modulates the voltage-dependence of Ca_v_1.4 channels thereby enhancing RBC responses (Haeseleer et al., 2004). Rod and cone synaptic function is markedly diminished in CaBP4 knockout mice (Haeseleer et al., 2004), and loss-of function mutations in CaBP4 cause congenital stationary night blindness and other visual disorders in humans (Littink et al., 2010; Bijveld et al., 2013). Potentially, light-dispersed Gα_t1_ may relieve UNC119-dependent constraint on the CaBP4 regulation of Ca_v_1.4 channels. Another lipid-independent interactor of UNC119 is RIBEYE, a major component of synaptic ribbons (Schmitz et al., 2000; Alpadi et al., 2008). This interaction recruits UNC119 to synaptic ribbons, and it may be essential for synaptic transmission at the rod ribbon synapse (Alpadi et al., 2008). It is not known if and how transducin modulates the binding of UNC119 to RIBEYE, but this may represent a potential pathway contributing to the synaptic effects of transducin translocation.

An interesting and possibly critical property of UNC119 is its ability to interact with heterotrimeric Gα_t1_β_1_γ_1_, promote dissociation of Gα_t1_ from Gβ_1_γ_1_, and release them from the membrane (Gopalakrishna et al., 2011). Analysis of the complex between UNC119 and the full-length Gα_t1_ by Small Angle X-ray Scattering (SAXS) and chemical crosslinking suggested an additional interface between the proteins involving the switch II region Gα_t1_, which overlaps with the Gβ_1_γ_1_ binding site (Cheguru et al., 2015). Thus, UNC119 apparently dissociates transducin subunits by disrupting and sterically occluding the Gβ_1_γ_1_-binding sites on Gα_t1_ (Cheguru et al., 2015). As a result, two species are produced, Gα_t1_GDP (or Gα_t1_GDP-UNC119 complex) and Gβ_1_γ_1_. Each of these species may now interact with new partners. In particular, Gα_t1_GDP is primed for interaction with Leu-Gly-Asn repeat-enriched (LGN) protein LGN/GPSM2 (Mochizuki et al., 1996). LGN/GPSM2 belongs to the class of G-protein modulators containing G-protein regulatory (GPR) or GoLoco motifs. GoLoco/GPR-proteins interact with and stabilize Gα subunits in a GDP-bound form, hence serving as guanine nucleotide dissociation inhibitors (GDIs; Natochin et al., 2000; Bernard et al., 2001; Blumer et al., 2007). LGN/GPSM2 is best known for its role in Gα-regulated positioning of a mitotic spindle during cell division (Lancaster and Knoblich, 2012; di Pietro et al., 2016). However, LGN/GPSM2 is also expressed in terminally differentiated photoreceptor cells, where it is localized to the IS/ST (Kerov et al., 2005; Nair et al., 2005). LGN/GPSM2 was shown to interact with endogenous Gα_t1_, and its role as a potential modulator of transducin trafficking has been proposed (Kerov et al., 2005). Evidence supporting this notion is starting to emerge (Bocchero et al., 2020). Interestingly, disruption of planar polarity mechanisms involving Gα_i3_ and LGN/GPSM2 collapsed the gradients of ribbon size and maximal synaptic Ca^2+^ influx in the inner hair cells (Jean et al., 2019), suggesting that Gα_t1_-LGN/GPSM2 signaling may also be involved in regulation of ribbon synapses in photoreceptors.

The synaptic regulation by translocated Gα_t1_ may be due to sequestration of Gβγ. In a well-characterized mechanism, the release of neurotransmitters at synapses of the central nervous system triggers negative feedback by activating presynaptic G protein-coupled receptors (GPCRs) and thereby generating free Gβγ. Gβγ in turn inhibits Ca^2+^ influx by binding directly to the α-subunits of presynaptic Ca_v_2.1 and Ca_v_2.1 channels (Catterall, 2011; Khan et al., 2013; Zamponi and Currie, 2013). In contrast, Ca_v_1.4 is not known to be regulated directly by Gβγ. A distinct mechanism of synaptic inhibition involves interactions between Gβγ subunits and the SNARE complex, specifically SNAP25 (Blackmer et al., 2001, 2005; Yoon et al., 2007). In cone photoreceptors, activation of presynaptic metabotropic glutamate receptor reduced synaptic transmission to horizontal cells via Gβγ/SNARE interactions (Van Hook et al., 2017). Furthermore, phosducin, an abundant Gβγ-binding protein in photoreceptors, was also implicated in regulation of synaptic transmission to RBCs, as the protein knockout resulted in a reduced sensitivity of ERG responses from RBCs in dark-adapted mice (Herrmann et al., 2010). These findings though were not confirmed in recordings from retinal slices, indicating that such experimental conditions may have abolished the phenotype (Long et al., 2013). It is possible that Gα_t1_ and/or phosducin sequester Gβγ liberated by activation of presynaptic GPCRs and thus enhance sensitivity of the rod-RBC synaptic transmission.

## Ric-8A in Photoreceptors: A GEF or a Chaperone?

In considering novel potential partners of Gα_t1_ in photoreceptor cells, one candidate, Ric-8A, was conspicuous. Resistance to inhibitors of cholinesterase 8 (Ric-8) proteins were originally discovered as positive regulators of G-protein signaling pathways (Miller et al., 1996, 2000). Subsequent studies demonstrated that Ric-8 proteins interact directly with the monomeric GDP-bound Gα subunits and act as non-GPCR guanine nucleotide exchange factors (GEFs; Tall et al., 2003). Two isoforms, Ric-8A and Ric-8B are encoded in vertebrate genomes. Each isoform regulates a particular subset of Gα subunits: Ric-8A interacts with Gα_i/t_, Gα_q_, and Gα_12/13_, whereas Ric-8B is selective for Gα_s_ (Klattenhoff et al., 2003; Tall et al., 2003; Von Dannecker et al., 2005; Nagai et al., 2010). The interaction of Ric-8 with GDP-bound Gα stimulates release of GDP, leading to the formation of a stable intermediate complex of Ric-8 and nucleotide-free Gα. Once Gα binds GTP, it dissociates from Ric-8, and thus the nucleotide-exchange cycle on Gα is completed (Tall et al., 2003). The GEF activity of Ric-8A opposing the GDI-activity of LGN/GPSM2 might be important to the role of Ric-8A in Gα-regulated positioning of mitotic spindle (Afshar et al., 2004; David et al., 2005; Tall and Gilman, 2005). Still, the interplay between Ric-8A and LGN/GPSM2 proteins in this process is poorly understood. More recently, Ric-8A attracted attention as a chaperone of Gα subunits (Papasergi et al., 2015). In cell-free translation systems, Ric-8A was required for the expression of properly folded Gα subunits, and co-expression of Ric-8 with Gα subunits in HEK293 cells and insect cells led to significant elevations in the expression levels of Gα subunits (Chan et al., 2011, 2013; Gabay et al., 2011). Although compelling evidence has been accumulated for both the GEF and chaperone function of Ric-8A *in vitro* and in cell cultures, little is known about specific pathways and systems regulated by Ric-8A *in vivo*, and it is often unclear which of the two Ric-8A functions dominates its biological effects. This is in part due to embryonic lethality of the Ric-8A knockout mice (Tonissoo et al., 2010; Gabay et al., 2011). Conditional knockouts of Ric-8A in differentiated neuronal populations and glial cells reveal apparent and severe phenotypes, yet the exact mechanisms of the Ric-8A deficiency underlying these phenotypes could not be discerned (Ma et al., 2012; Ruisu et al., 2013). Targeted disruption of Ric-8A expression in mouse B-cells led to a loss of Gα_i_ and Gα_q_ and caused severe humoral immunodeficiency, a phenotype consistent with the chaperone function of the protein (Boularan et al., 2015).

**FIGURE 1:**
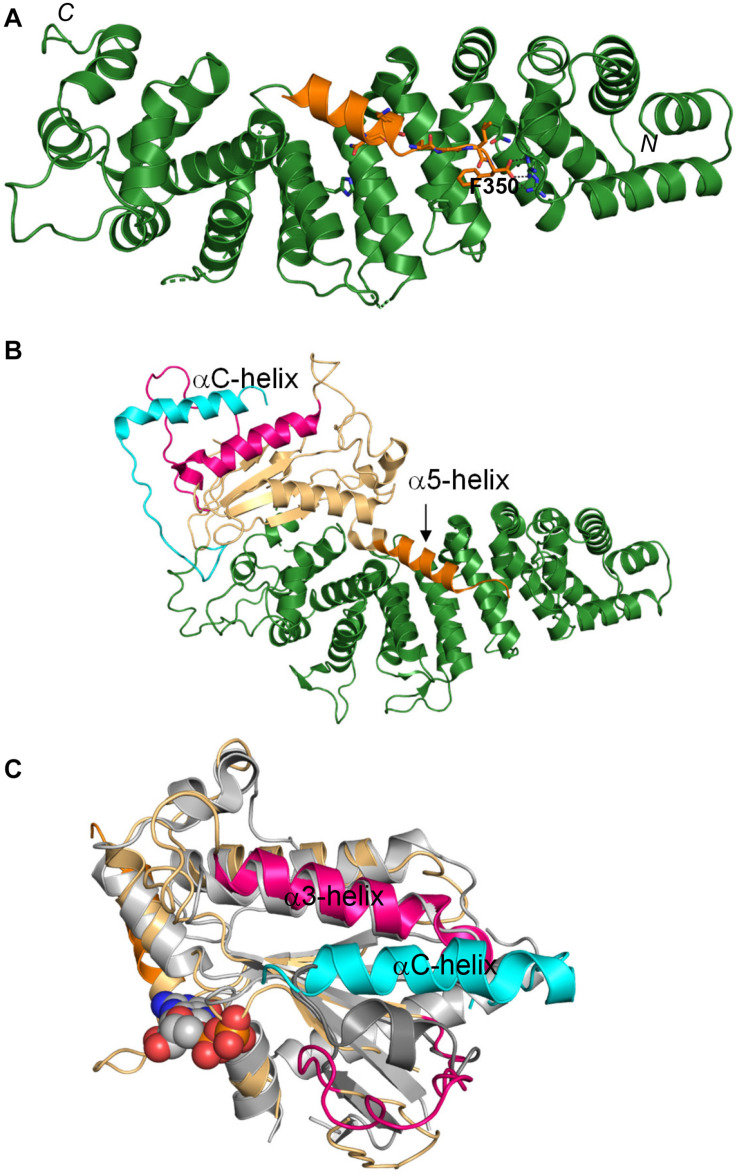
Interaction of Ric-8A with Gα. **(A)** The α5-helix of Gα_t1_ (orange) bound to the concave surface of the armadillo core domain of Ric-8A (green; based on PDB 6N85). The C-terminal F350 of Gα_t1_ forms multiple contacts with Ric-8A. **(B)** Crystal structure of the complex of Ric-8A with Gα_i_ (PDB 6TYL). Only the Ras-like domain of Gα_i_ is resolved in the structure (wheat). The a5-helix of Gα_i_ (orange) is dislodged from the β-sheet core. The C-terminal helix of Ric-8A (αC, cyan) interacts with the switch II/α3 helix of Gα_i_ (pink). **(C)** Top view of the Gα_i_ complex with Ric-8A. Only the C-terminus of Ric-8A is shown for clarity. The αC-helix of Ric-8A (cyan) interacts with the switch II/a3-helix region (pink) of Gα_i_ (wheat). This interaction may facilitate the binding of GTP to Gα bound to Ric-8A. The GPCR-bound structure of the engineered minimized Gα (gray, GDP – spheres, and PDB 5G53) is superimposed with Gα_i_ to indicate position of the nucleotide binding site.

Growing evidence for the important biological roles of Ric-8 spurred the interest in understanding the molecular and structural basis underlying its activities. This work culminated in solution of the atomic structures initially of Ric-8A in complex with a Gα_t__1_ mimetic, and subsequently in complex with the full-length Gα subunits (Srivastava and Artemyev, 2019; Srivastava et al., 2019; Zeng et al., 2019; McClelland et al., 2020; Seven et al., 2020). The structure of the active nearly full length Ric-8A1-492 revealed two main domains of Ric-8A: an armadillo-like core (residues about 1–426) and an unstructured C-terminal tail (residues 427–492; Srivastava et al., 2019). The armadillo-like core of Ric-8A is a mixture of canonical ARM repeats and ARM-related HEAT repeats folded into a ribbon-like superhelix featuring a concave surface (Srivastava et al., 2019; Zeng et al., 2019). The concave surface of Ric-8A forms an extensive interface with the Gα_t1_ C-terminus (Figure 1A). Critical contacts between Ric-8A and the Gα C-terminus are made by the very C-terminal residue F350 of Gα_t1_ (Figure 1A; Srivastava et al., 2019). Mutations interfering with the interaction network of the C-terminal residue of Gα ablate the binding of Ric-8A to folded GαGDP (Srivastava et al., 2019), as well as disrupt the chaperone activity of Ric-8A (Seven et al., 2020). As a GEF, Ric-8A interacts with the folded GαGDP and induces partial unfolding of the latter accompanied by the large dislocation of the Gα C-terminal α5-helix from the β-sheet core of the Ras-like domain, disorganization of the nucleotide-binding site and release of GDP (Srivastava and Artemyev, 2019; McClelland et al., 2020; Seven et al., 2020; Figure 1B). As a chaperone of nascent Gα, Ric-8A would interact with a partially folded intermediate of Gα in which the α5-helix has not yet assumed its position with the β-sheet cradle (Seven et al., 2020). The Gα_t1_ C-terminal region is unstructured as a part of MBP-fusion protein, but it forms the α5-helix upon binding to Ric-8A (Srivastava et al., 2019). Therefore, the chaperone activity of Ric-8A may involve folding of the Gα C-terminal region into an α5-helix and stabilization of the β-sheet core of the Ras-like domain thereby preparing Gα for the first time GTP-binding event (Srivastava et al., 2019; McClelland et al., 2020; Seven et al., 2020). Upon binding of GTP, Gα is released from Ric-8A and the α5-helix replaces Ric-8A in stabilizing the β-sheet core of Gα. The GTP-binding site in the Ric-8A/Gα complex is disorganized to a greater extent compared to that in the GPCR/Gαβγ complexes (McClelland et al., 2020; Seven et al., 2020; Srivastava and Artemyev, 2020). Remarkably, the distal portion of the C-terminal tail of Ric-8A forms a unique smaller secondary interface with the switch II/α3-helix region of Gα, which appears to assist GTP-binding (Srivastava and Artemyev, 2019; McClelland et al., 2020; Seven et al., 2020; Figure 1C). Altogether, the structures of the Ric-8A/Gα complex are consistent with both proposed functions of Ric-8A, as a GEF and a chaperone. Specific function of Ric-8A would have to be determined in the context of a cellular activity under investigation.

**FIGURE 2:**
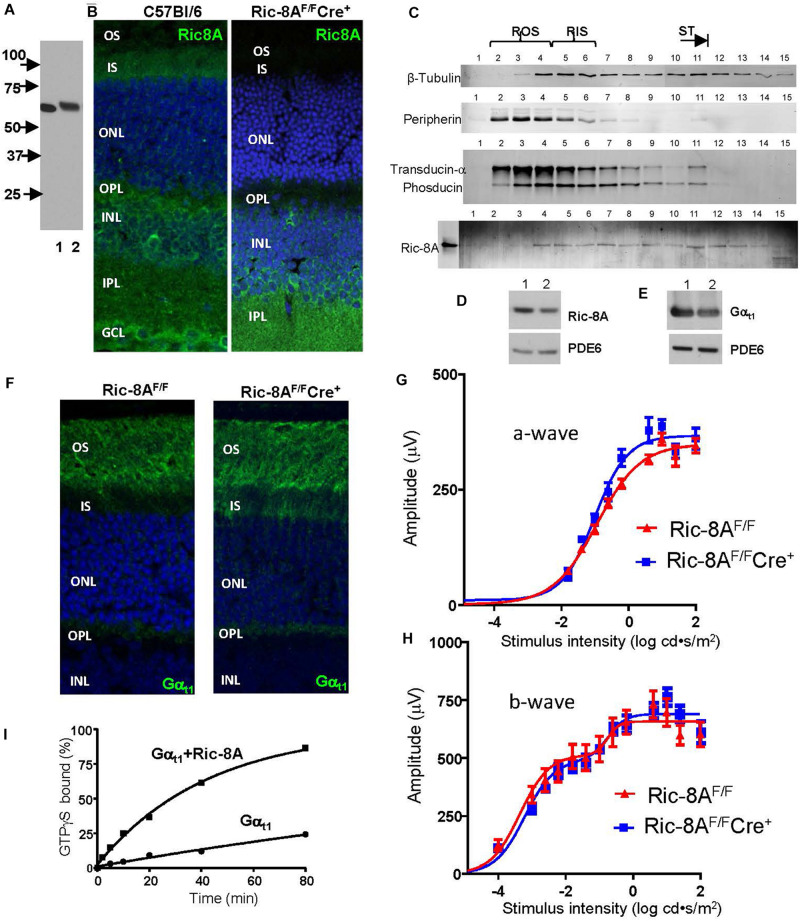
Expression of Ric-8A in the retina and conditional knockout of the protein in mouse rods. **(A)** Western blot with anti-Ric-8A monoclonal antibody 3E1; lane 1 – recombinant human Ric-8A (50 ng), lane 2 – C57Bl retina homogenate (50 μg). **(B)** Immunofluorescence (IF) staining of mouse retina cryosections with 3E1 monoclonal antibody. Ric-8A is present throughout the retina in 2-month old WT mice and absent in the inner segment (IS), outer nuclear layer (ONL), and outer plexiform layer (OPL) in 2-month old *Ric-8A^*F/F*^Cre^+^* mice. INL, inner nuclear layer; IPL, inner plexiform layer, GCL, ganglion cell layer; and blue, To-Pro3 nuclear stain. **(C)** Western blot analysis of tangential sections of WT retina indicates the presence of Ric-8A in the IS (approx. lanes 5–6), the inner compartments of rods (approx. lanes 7–11), and the bipolar cell layer (approx. lanes 12–15). Ric-8A is largely excluded from the outer segment (OS; approx. lanes 2–4). Lane 0 – recombinant Ric-8A. **(D, E)** Western blot analyses of equal fractions of total retina extract from control 2-month old *Ric-8A^*F/F*^* mice (lane 1) and littermate *Ric-8A^*F/F*^Cre^+^* mice (lane 2) with anti-Ric-8A antibody 3E1 **(D)** and anti-Gα_t1_ K-20 antibody (SCBT; **E)**. PDE6 – loading control. The bands were quantified with ImageJ. From three similar experiments, the average reductions of Ric-8A and Gα_t1_ in the *Ric-8A^*F/F*^Cre^+^* retina were 36 ± 3% and 22 ± 4%, respectively. **(F)** IF staining of retina cryosections from dark-adapted mice with anti-Gα_t1_ K-20 antibody. Gα_t1_ is localized mainly to the rod OS in 2-month old *Ric-8A^*F/F*^Cre^+^* mice. **(G, H)** a-wave amplitudes and b-wave amplitudes measured from recordings of dark-adapted mice. Points represent the mean ± SEM (*n* = 6, left and right eyes from 3 mice of each genotype). Curves represent fits from single **(G)** or double **(H)** sigmoidal functions. For each flash strength, there were no statistically significant differences (adjusted *P* value > 0.05) between *Ric-8A^*F/F*^Cre^+^* and control mice. **(I)** Kinetics of GTPγS binding to chimeric Gα_t1_ (Skiba et al., 1996; Natochin et al., 1998) alone (1 μM) and in the presence of Ric-8A (3 μM). Representative experiment. For *n* = 3 experiments, the *k*_*app*_ values are: Gα_t1_ 0.005 ± 0.001 min^– 1^ and Gα_t1_+Ric-8A 0.025 ± 0.002 min^– 1^; unpaired *t*-test.

Given the high rate of transducin synthesis and transport in photoreceptors (Frederick et al., 2020), and the likely existence of the quality control chaperone mechanism, we first investigated expression of Ric-8A in retina and photoreceptor cells. In homogenates of C57Bl/6 mouse retina specific monoclonal Ric-8A antibody 3E1 recognized a single band of the predicted MW of 60 kDa (Figure 2A; Gabay et al., 2011). Immunofluorescence staining of cryosections of the C57Bl/6 retina with 3E1 antibody revealed that Ric-8A is distributed throughout the retina, including in the IS, OPL, INL, and IPL (Figure 2B). Expression of Ric-8A in the photoreceptor IS/ST was also confirmed by Western blot analysis of tangential retina sections (Figure 2C; Sokolov et al., 2002). The retinal distribution of Ric-8A suggested that it can serve as a chaperone for newly synthesized Gα_t1_ and/or a GEF for light-translocated Gα_t1_ in rods, as well as chaperone/GEF for Gα_o_ in RBCs. Gα_o_ is abundant in RBCs, where it mediates signaling via the mGluR6 cascade, which couples a decrease in glutamate levels in the synaptic cleft with opening of the TRPM1 cation channels in the dendritic tips of RBCs (Dhingra et al., 2000, 2002). To test the former hypothesis, we generated a mouse model with conditional knockout of Ric-8A in rods. To achieve specific deletion of Ric-8A in rods, *Ric-8A^*F/F*^* mice in which exons 2–4 of the gene are floxed (Ma et al., 2012) were crossed to *iCre-75^+/–^* mice, in which expression of Cre is driven by a 4-kb mouse rod opsin promoter (Li et al., 2005). The *iCre-75^+/–^* driver strain is commonly used for RP-specific conditional KO, as it provides for robust and uniform expression of Cre in rods (Li et al., 2005; Lai et al., 2013; Sundermeier et al., 2014; He et al., 2016). Immunofluorescence staining of retina cryosections from *Ric-8A^*F/F*^Cre^+^* mice confirmed robust expression of Cre (Supplementary Figure 1) and deletion of Ric-8A in mutant rods (Figure 2B and Supplementary Figure 2). Western blot analysis of Ric-8A in dark-adapted *Ric-8A^*F/F*^Cre^+^* retinas indicated that the protein level is reduced by ∼36% (Figure 2D). The extent of reduction in the protein level of Ric-8A in the entire retina is consistent with the localization of a major fraction of the protein to the inner retina. Surprisingly, the ablation of Ric-8A expression in rods caused only a modest ∼22% reduction in the protein level of Gα_t1_ (Figure 2E). Furthermore, the majority of Gα_t1_ was properly targeted to the OS in the absence of Ric-8A (Figure 2F). Supporting the functional folding of transducin in rods lacking Ric-8A, the a- and b-wave ERG responses of mutant mice were comparable to those from control mice (Figures 2G,H). Thus, our results indicate that although Ric-8A may slightly increase the abundance of Gα_t1_ in rods, the proper folding of Gα_t1_ in the absence of Ric-8A proceeds efficiently enough to support the photoreceptor function. Although mouse rods lacking Ric-8A may express Ric-8B, the latter isoform does not interact with Gα_t1_ (Papasergi et al., 2015; Srivastava et al., 2019). One caveat to this conclusion needs to be noted. It cannot be excluded that Ric-8A is a catalytic chaperone that can efficiently assist folding of Gα_t__1_ even when present in trace amounts. Trace amounts of Ric-8A in mutant rods may result if the Ric-8A protein expressed prior to the gene excision persists for a long time or if the gene excision is incomplete.

Nevertheless, it is possible that the main function of Ric-8A in rods is linked to its GEF activity. We confirmed that Ric-8A is a GEF for the Gα_t_ similarly as it is for other members of the Gα_i_ family by measuring the kinetics of GTPγS-binding to a transducin-like chimeric Gα_t1_GDP in the presence or absence of Ric-8A. The apparent rate of the nucleotide exchange was increased by ∼5-fold in the presence of Ric-8A (Figure 2I). Future studies are needed to investigate functional significance of the Ric-8A GEF activity toward Gα_t1_.

## Discussion

The structures of the Ric-8A complexes with Gα subunits reveal the molecular underpinnings of its chaperone and GEF activities (Srivastava and Artemyev, 2019; Srivastava et al., 2019; McClelland et al., 2020; Seven et al., 2020). Depending on the Ric-8A function in a given system, the structures can represent either the GEF complex intermediate with an empty-pocket for nucleotide binding or the folding intermediate of Gα during its biosynthesis. The Ric-8A/Gα complexes reveal two remarkable features: a large displacement of the α5-helix of Gα from the β-sheet cradle of the Ras-like domain, and a unique interaction of the C-terminal helix of Ric-8A with the switch II/α3-helix region of Gα (Srivastava and Artemyev, 2019, 2020; McClelland et al., 2020; Seven et al., 2020). The interaction of Ric-8A with the C-terminus/α5-helix of Gα is central to both the GEF and the chaperone activity. When Ric-8A acts as a GEF, this interaction initiates GDP-release, just like GPCRs cause GDP-release during activation of heterotrimeric G-proteins. When Ric-8A acts as a chaperone, this interaction induces the folding of the α5-helix outside the β-sheet cradle of partially folded Gα (Srivastava et al., 2019; McClelland et al., 2020). The interaction of the C-terminal helix of Ric-8A with the switch II/α3-helix region of Gα likely promotes GTP-binding to Gα thereby concluding either the GEF or the chaperone cycle of Ric-8A (Srivastava and Artemyev, 2019, 2020; McClelland et al., 2020; Seven et al., 2020). We demonstrated that Ric-8A is expressed throughout the retina. Thus, retina represents an excellent opportunity to dissect the roles of Ric-8A. The conditional knockout of Ric-8A in mouse rods argues against its role as an essential chaperone of Gα_t1_. Yet, as a GEF, Ric-8A may play roles in transducin trafficking and/or modulation of the rod-RBC synaptic transmission. The role of Ric-8A in RBCs and its potential influence on the abundance of Gα_o_ and the mGluR6-mediated cascade remains to be investigated. Gα_q__/11_ is the Gα subfamily that is most stringently dependent on Ric-8A as a chaperone (Gabay et al., 2011). Therefore, defining role of Ric-8A in intrinsically photosensitive retinal ganglion cells and the Gα_q_-mediated melanopsin signaling cascade is of particular interest (Do and Yau, 2010; Schmidt et al., 2011).

## Data Availability Statement

All datasets presented in this study are included in the article/Supplementary Material.

## Ethics Statement

The animal study was reviewed and approved by the University of Iowa Animal Care and Use Committee.

## Author Contributions

NA wrote the manuscript. All authors contributed to the study, read and approved the manuscript.

## Conflict of Interest

The authors declare that the research was conducted in the absence of any commercial or financial relationships that could be construed as a potential conflict of interest.
