# Supplementary material for: Transducin Partners Outside the Phototransduction Pathway
Source: Front Cell Neurosci. 2020 Oct 14;14:589494. doi: 10.3389/fncel.2020.589494 (PMC7591391; doi:10.3389/fncel.2020.589494)
Supplement: Supplementary file 1 [file Data_Sheet_1.docx]

**Supplementary material**

**Animals**

All experimental procedures involving the use of mice were carried out in accordance with the NIH guidelines and the protocol approved by the University of Iowa Animal Care and Use Committee. A conditional Ric8A KO mouse was generated from double conditional *Ric8A^F/F^;Ric8B^F/F^* conditional *Ric8A^F/F^;Ric8B^F/F^* mice obtained in laboratory of Dr. Z. Huang (Univ. of Wisconsin). In the *Ric8A^F^* conditional allele, exons 2-4 are floxed (Ma et al., 2012). *Ric8A^F/F^;Ric8B^F/F^* mice were crossed to C57Bl/6 mice, and the double heterozygous offspring (*Ric8A^F/+^; Ric8B^F/+^*) were mated to obtain homozygous *Ric8A^F/F^* and *Ric8B^F/F^* mice. To achieve RP-specific deletion of Ric8A, *Ric8A^F/F^* mice were crossed to *iCre-75^+/-^* mice, in which expression of Cre is driven by a 4-kb mouse rod opsin promoter (Li et al., 2005). *Ric8A^F/+^Cre^+^* and *Ric8A^F/F^* mice were crossed to obtain *Ric8A^F/F^Cre^+^* mice with the Ric8A deletion in RPs.

**Immunoblot analysis**

Total mouse retinal homogenates were obtained by solubilization of two retinas in 100 µl of 10% SDS-Na using brief sonication. Protein concentrations were determined using the DC Protein Assay (Bio-Rad) with bovine serum albumin dissolved in 10% SDS-Na serving as the standard. Samples of retinal homogenates were analyzed by immunoblotting using the following primary antibodies (1:1000 dilution): rod Gα_t1_ K-20 (Santa Cruz Biotechnology), Ric-8A monoclonal 3E1 (Sigma-Aldrich), peripherin RDH-A1, β-tubulin T0198 (Sigma-Aldrich), and phosducin antibody as in (Sokolov et al., 2004). Levels of Gα_t1_ and Ric-8A were quantified by measuring the integrated densities of the bands using ImageJ.

**Immunofluorescence**

Mice were dark-adapted for at least 12 hrs. The mice were euthanized by CO_2_ asphyxiation. Mouse eyeballs were enucleated, poked through the cornea with a 21-gauge needle, and fixed in 4% paraformaldehyde in phosphate-buffered saline (PBS) for 1 hr at 25 °C. For dark-adapted mice, euthanasia, enucleation of eyeballs and initial fixation were all performed under dim red light. After this initial fixation, the eyeballs were cut to remove the cornea and lens, and the eyecups were fixed in 4% paraformaldehyde in PBS for an additional 3 hrs at 25 °C. The eyecups were then transferred to a 1:1 solution of 30% sucrose in PBS and 4% paraformaldehyde in PBS for 1 hr at 25 °C. Finally, the eyecups were submersed in a 30% sucrose solution in PBS and left at 4°C overnight. The next day, eyecups were embedded in tissue freezing medium (TBS) and frozen into cryo blocks on dry ice. Cryo blocks were subsequently sectioned (10 µm, Microm HM 505E) and stored at -80 °C until use. Before staining, sections were warmed up to room temperature overnight. On the following day, sections were first incubated in 0.1% Triton X-100 in PBS for 30 min, followed by a 1 hr incubation with either a rabbit anti-rod Gα_t1_ antibody K-20 (1:1000) (Santa Cruz Biotechnology), Ric-8A monoclonal 3E1 antibody (1:250)(Sigma-Aldrich) or monoclonal (clone 7.23) anti-Cre recombinase antibody (1:100) Biolegend). The sections were then stained with either goat anti-rabbit AlexaFluor 546 or goat anti-mouse AlexaFluor 488 secondary antibodies (Molecular Probes) (1:1000) for 1 hr. All sections were subsequently counterstained with TO-PRO3 nuclear stain. The stained sections were visualized using a Zeiss LSM 510 confocal microscope.

**Tangential retina sectioning**

Subcellular distribution of proteins in rod photoreceptors was determined by immunoblotting of serial tangential sections of frozen retina, according to the original procedure (Sokolov et al., 2002), which was modified for sectioning mouse retina (Song and Sokolov, 2009). The latest version of this protocol is provided below.

All experiments involving the use of mice were carried out according to protocols approved by the West Virginia University Animal Care and Use Committee. Mice were euthanized, and their eyes were enucleated and dissected. The dissection was conducted under a surgical microscope under standard illumination for light adapted eyes or infrared illumination for dark adapted eyes. During the dissection, the cornea and the lens were removed, and a 2mm trephine was used to excise a circular punch-out from the posterior hemisphere. The retina attached to the punch-out was flattened inside of an acrylic cylindrical chamber holding a glass capillary array that was connected to a syringe (GCA 09/32/25/0/20 LM, BURLE Electro-Optics, Sturbridge, MA). The chamber was filled with DMEM media, and the punch-out was transferred into the chamber. The retina was gently pulled away from the retinal pigmented epithelium and choroid and laid “photoreceptor-down” onto a supporting piece of polyvinylidene difluoride (PVDF) membrane that was pre-wetted with DMEM media. Gentle suction was applied from underneath the glass capillary array to slowly remove all media from the chamber. Retina/PVDF was clamped between two glass slides separated by 0.5 mm spacers, and frozen on dry ice. Frozen retina was sectioned using a Leica CM1850 Cryostat. To create a mound for the glass slide, OCT compound was frozen to the specimen holder and cut to generate a surface aligned with the cutting plane. The top glass slide and the spacers were removed from the frozen Retina/PVDF. The base glass slide with retina/PVDF was mounted onto the OCT by applying water drops to the sides of the glass slide. To remove folded edges, retina/PVDF was trimmed along the entire perimeter, and the remaining fragment was sectioned at 10 µm. After the microtome blade made initial contact with the tissue and cut the first continuous section, the parts of the tissue not included in this section were trimmed away. From that point on, serial sections were collected by picking up each section with a plastic toothpick and transferring them into 50*μ*L of SDS-PAGE sample buffer (6 M urea, 125 mM Tris-HCl, pH 6.8, 4% SDS, bromophenol blue, and 5% β-mercaptoethanol). Sections were stored at -80ᶱC until their protein content was analyzed by Western blotting. The collection ended when the microtome knife began to cut PVDF. In each set of sections, the alignment of the retinal layers was assessed using dot blotting with anti-rhodopsin. The set was considered acceptable if the marker of photoreceptor outer segments layer, rhodopsin, appeared only in the last 3-4 sections adjacent to PVDF membrane.

**Electroretinography (ERG)**

ERG recordings were obtained using the Espion E^3^ system (Diagnosys LLC, Lowell, MA). After overnight dark adaptation, five- to six-weeks old mice were prepared for ERG recording under dim red light. Mice (5-6 week old, males and females) were anesthetized by intraperitoneal injection of a ketamine/xylazine mixture (100 and 10 mg/kg respectively). The pupils were dilated by applying a drop of 1% tropicamide, followed by a drop of 2.5% phenylephrine hydrochloride. ERGs were recorded simultaneously from the corneal surface of each eye using gold ring electrodes (Diagnosys), with a needle electrode placed on the back of the head serving as reference. Another needle electrode placed near the tail served as ground. A drop of Hypromellose 2.5% Ophthalmic Demulcent Solution was placed on the corneal surface to ensure electrical contact and to prevent eyes from drying and cataract formation. Body temperature of mice was maintained at 37°C using the system's heating pad. Mice were placed in a Ganzfeld stimulator chamber (ColorDome; Diagnosys) for delivery of stimuli, and the mouse head and electrode positioning were monitored on the camera attached to the system. ERG responses were evoked in mice by a series of flashes ranging from 0.0001 to 100 cd∙s/m^2^. Responses to 6 sweeps were averaged for dim flashes up to 0.6 cd∙s/m^2^, 2 sweeps were averaged for 4 cd∙s/m^2^, and responses to brighter flashes were recorded without averaging. Inter sweep intervals for flashes with increasing strength were increased from 10 sec to 60 sec to allow full recovery from preceding flashes. The data were fitted to single or double sigmoid functions as described previously (Herrmann et al., 2010) using GraphPad Prizm 8 software. The ERG datasets were analyzed by two-way ANOVA followed up by Bonferroni test. For each flash strength, there were no statistically significant differences (adjusted P value >0.05) between *Ric-8A^F/F^Cre^+^* and control mice.

**GTPγS-binding assay**

Binding reactions were started with the addition of 5 µM [^35^S]GTPγS to chimeric Gα_t1_ (1 µM) alone and in the presence of Ric-8A (3 μM) at 25ºC. Aliquots of 15 µl were withdrawn at the indicated times, mixed with 1 ml ice-cold 20 mM Tris-HCl (pH 8.0) buffer containing 130 mM NaCl, 2 mM MgSO_4_, and 1 mM GTP, passed through Whatman cellulose nitrate filters (0.45 µm), and washed three times with 3 ml of the same buffer without GTP. The filters were dissolved in 5 ml of a xylene-based 3a70B counting cocktail (RPI Corp.) and [^35^S]GTPγS was measured in a liquid scintillation counter (Natochin and Artemyev, 2003). The k_app_ values for the binding reactions were calculated by fitting data with equation %GTPγS bound=100(1-e^-kt^).

**References for supplemental material**

Herrmann, R., Lobanova, E.S., Hammond, T., Kessler, C., Burns, M.E., Frishman, L.J., et al. (2010). Phosducin regulates transmission at the photoreceptor-to-ON-bipolar cell synapse. *The Journal of neuroscience : the official journal of the Society for Neuroscience* 30(9)**,** 3239-3253. doi: 10.1523/JNEUROSCI.4775-09.2010.

Li, S., Chen, D., Sauve, Y., McCandless, J., Chen, Y.J., and Chen, C.K. (2005). Rhodopsin-iCre transgenic mouse line for Cre-mediated rod-specific gene targeting. *Genesis* 41(2)**,** 73-80. doi: 10.1002/gene.20097.

Ma, S., Kwon, H.J., and Huang, Z. (2012). Ric-8a, a guanine nucleotide exchange factor for heterotrimeric G proteins, regulates bergmann glia-basement membrane adhesion during cerebellar foliation. *J Neurosci* 32(43)**,** 14979-14993. doi: 10.1523/JNEUROSCI.1282-12.2012.

Natochin, M., and Artemyev, N.O. (2003). A point mutation uncouples transducin-alpha from the photoreceptor RGS and effector proteins. *J Neurochem* 87(5)**,** 1262-1271. doi: 10.1046/j.1471-4159.2003.02103.x.

Sokolov, M., Lyubarsky, A.L., Strissel, K.J., Savchenko, A.B., Govardovskii, V.I., Pugh, E.N., Jr., et al. (2002). Massive light-driven translocation of transducin between the two major compartments of rod cells: a novel mechanism of light adaptation. *Neuron* 34(1)**,** 95-106.

Sokolov, M., Strissel, K.J., Leskov, I.B., Michaud, N.A., Govardovskii, V.I., and Arshavsky, V.Y. (2004). Phosducin facilitates light-driven transducin translocation in rod photoreceptors. Evidence from the phosducin knockout mouse. *J Biol Chem* 279(18)**,** 19149-19156. doi: 10.1074/jbc.M311058200.

Song, H., and Sokolov, M. (2009). Analysis of protein expression and compartmentalization in retinal neurons using serial tangential sectioning of the retina. *J Proteome Res* 8(1)**,** 346-351. doi: 10.1021/pr800631d.

**Supplemental Figure 1**. Immunofluorescence staining of mouse retina cryosection from 2-month old *Ric-8A^F/F^Cre^+^* animal with monoclonal anti-Cre recombinase antibody (clone 7.23, Biolegend) (green); blue - To-Pro3 nuclear stain. Outer segment layer (OS), inner segment layer (IS), outer nuclear layer (ONL) and outer plexiform layer (OPL) in 2-month old *Ric-8A^F/F^Cre^+^* mice. INL - inner nuclear layer.

**Supplemental Figure 2**. Immunofluorescence staining of mouse retina cryosections with 3E1 monoclonal antibody as in Figure 2B. Insets show magnified areas at the IS/ONL border.
